# Epidemiological characteristics of leukemia in China, 2005–2017: a log-linear regression and age-period-cohort analysis

**DOI:** 10.1186/s12889-023-16226-1

**Published:** 2023-08-28

**Authors:** Kangqian Lin, Huaimiao Jia, Miao Cao, Tongtong Xu, Zuhai Chen, Xi Song, Yingfang Miao, Teng Yao, Chenxian Dong, Jianjiang Shao, Heng Guo, Yunhua Hu, Yizhong Yan

**Affiliations:** 1 Department of Preventive Medicine, School of Medicine, Shihezi University, Shihezi, Xinjiang China; 2 Shihezi Center for Disease Control and Prevention, Shihezi, Xinjiang China; 3 Key Laboratory of Preventive Medicine, Shihezi University, Shihezi, Xinjiang China; 4 Key Laboratory of Xinjiang Endemic and Ethnic Diseases (Ministry of Education), School of Medicine, Shihezi University, Shihezi, Xinjiang China; 5 Key Laboratory for Prevention and Control of Emerging Infectious Diseases and Public Health, The Xinjiang Production and Construction Corps, Xinjiang, China

**Keywords:** Leukemia, Incidence, Mortality, Age-period-cohort model, Joinpoint regression model

## Abstract

**Background:**

Leukemia is a threat to human health, and there are relatively few studies on the incidence, mortality and disease burden analysis of leukemia in China. This study aimed to analyze the incidence and mortality rates of leukemia in China from 2005 to 2017 and estimate their age-period-cohort effects, it is an important prerequisite for effective prevention and control of leukemia.

**Methods:**

Leukemia incidence and mortality data from 2005 to 2017 were collected from the Chinese Cancer Registry Annual Report. Joinpoint regression model was used to estimate the average annual percentage change (AAPC) and annual percentage change (APC) response time trend. Age-period-cohort model was constructed to analyze the effects of age, period and cohort.

**Results:**

The age-standardized incidence rate of leukemia was 4.54/100,000 from 2005 to 2017, showed an increasing trend with AAPC of 1.9% (95% CI: 1.3%, 2.5%). The age-standardized mortality rate was 2.91/100,000, showed an increasing trend from 2005 to 2012 with APC of 2.1% (95%CI: 0.4%, 3.9%) and then a decreasing trend from 2012 to 2017 with APC of -2.5% (95%CI: -5.3%, 0.3%). The age-standardized incidence (mortality) rates of leukemia were not only higher in males than that in females, but also increased more rapidly. The incidence of leukemia in rural areas was lower than in urban areas, but the AAPC was 2.2 times higher than urban areas. Children aged 0–4 years were at higher risk of leukemia. The risk of leukemia incidence and mortality increased with age. The period effect of leukemia mortality risk showed a decreasing trend, while the cohort effect showed an increasing and then decreasing trend with the turning point of 1955–1959.

**Conclusions:**

The age-standardized incidence rate of leukemia in China showed an increasing trend from 2005 to 2017, while the age-standardized mortality rate increased first and then decreased in 2012 as a turning point. Differences existed by gender and region. The risk of leukemia incidence and mortality increased accordingly with age. The risk of mortality due to leukemia gradually decreased from 2005 to 2017. Leukemia remains a public health problem that requires continuous attention.

## Introduction

Malignant neoplasm is the leading cause of death from disease in developed countries and the second cause in developing countries worldwide [1, 2]. In a latest report from the International Agency for Research on Cancer, there were an estimated 19.3 million new cancer cases and nearly 10 million cancer deaths worldwide in 2020 [3]. It seriously affects and threatens human health, and has become one of the most prominent major public health problems worldwide [4].

Leukemia is a common malignancy of the hematologic system, a category of malignant clonal diseases of hematopoietic stem cells characterized by uncontrolled malignant proliferation of mature leukocytes and their precursors in the blood and bone marrow [5]. It has a poor prognosis, with the 5-year survival rate of leukemia patient still ≤ 50% [6]. The Cancer Statistics Report showed that the number of new leukemia cases globally reached 475,000 in 2020, an 84.82% increase from 2000, and the number of deaths from leukemia globally reached 312,000 in 2020, a 60.0% increase from 2000 [3, 7]. China accounted for 62,000 deaths due to leukemia in 2020, 19.87% of the global leukemia deaths [1]. Leukemia treatment is extremely expensive and difficult to cure, causing great hardship for patients and families [8].

Leukemia is a part of the United Nations’ third Sustainable Development Goals, which aims to cut premature mortality from non-communicable diseases by one third by 2030 [9]. Tracking changes in the burden of leukemia could provide relevant data for better policy development. Considering the relatively few studies on leukemia incidence, mortality and disease burden analysis in China. The study collected national, gender-specific, region-specific leukemia incidence and mortality data from the Chinese Cancer Registry Annual Report from 2008 to 2020. Analyzed the trends of leukemia incidence and mortality using Joinpoint regression, and analyzed the effects of age, period, and cohort effects on the risk of leukemia incidence and mortality using an age-period-cohort model. In order to produce important basic data for the control of leukemia in China, to provide a scientific basis for leukemia prevention and treatment.

## Methods

### Data source

Data on incidence (mortality) rate and age-standardized incidence (mortality) rate (ASIR, ASMR) of leukemia in China from 2005 to 2017 (based on standard Chinese population in 2000) [10] were obtained from the Chinese Cancer Registry Annual Report, 2008–2020 [11–23]. Cases of leukemia are identified according to the International Classification of Diseases, 10th Revision (ICD-10, C91-95, D45-47) [24]. The classification of urban and rural areas in this study was based on the classification criteria of the Chinese Cancer Registry Annual Report: urban areas were classified as cities above prefecture level, and rural areas were classified as counties and county-level cities.

The raw data were collected from cancer registries in 31 provinces (autonomous regions and municipalities) and Xinjiang Production and Construction Corps. The data were reviewed, evaluated, collated and analyzed according to the requirements of the Chinese guideline cancer registration and the standards of International Agency for Research on Cancer/International Association of Cancer Registries [25].

### Construction of model

#### Joinpoint regression model

Trend of leukemia incidence and mortality was performed using Joinpoint regression models to calculate average annual percentage change (AAPC) and annual percentage change (APC) with their 95% confidence intervals (95% CI). The model creates a segmented regression based on the temporal characteristics of the disease distribution, divides the study time into different intervals through multiple connection points, and fits and optimizes the trends in each interval to evaluate the characteristics of specific disease changes in different intervals [26, 27]. The Monte Carlo permutation test was used to determine the number of connection points, the location of each connection point and the corresponding p-value, with a test level of α = 0.05 (two-sided test). Joinpoint regression models are linear and log-linear models, and the log-linear model is generally chosen when analyzing population-based trends in cancer incidence and mortality [28]:

$${\text{E}}[y|x]=\mathop e\nolimits^{{\mathop \beta \nolimits_{0} +\mathop \beta \nolimits_{1} x+\mathop \delta \nolimits_{1} \mathop {(x - \tau }\nolimits_{1} \mathop )\nolimits^{+} + \cdots +\mathop \delta \nolimits_{k} (x - \mathop \tau \nolimits_{k} )}}$$

Where *e* is the natural base, *k* indicates the number of turning points, $\mathop \tau \nolimits_{k}$indicates the unknown turning points, $\mathop \beta \nolimits_{0}$is the invariant parameter, $\mathop \beta \nolimits_{1}$is the regression coefficient, $\mathop \delta \nolimits_{k}$indicates the regression coefficient of the segment function in paragraph *k*. When $\mathop {(x - \tau }\nolimits_{k} \mathop )\nolimits^{{}}$> 0, $\mathop {(x - \tau }\nolimits_{1} \mathop )\nolimits^{+} =x - \mathop \tau \nolimits_{k}$, otherwise $\mathop {(x - \tau }\nolimits_{1} \mathop )\nolimits^{+} {\text{=}}0$.

Calculation formula of APC: $APC=\left( {{e^{\beta 1}}-{\text{1}}} \right) \times {\text{100}}$

Calculation formula of AAPC: ${\text{AAPC}}=\left[ {\exp \left( {\sum {{w_i}{\beta _i}} /\sum {{w_i}} } \right)-1} \right] \times {\text{100}}$

Where${\beta _1}$is the regression coefficient, ${w_i}$is the width of the interval span (i.e., the number of years included in the interval) for each segmentation function, and${\beta _i}$is the regression coefficient corresponding to each interval.

#### Age-period- cohort model

To analyze the effects of age, period, and cohort on leukemia incidence and mortality. Used 5 years as one age period, 0–89 years were divided into 18 age periods. The Poisson log-linear model was used to evaluate the age-period cohort model by solving the intrinsic estimator, and the model fitted goodness of scale was evaluated comprehensively using the red pool information criterion. The formula is expressed as

$$\log \left( {{\lambda _{apc}}} \right)={\alpha _a}+{\beta _p}+{\gamma _c}{\text{+}}\varepsilon $$

Where $\alpha$,$\beta$and$\gamma$are age, period and cohort effects, respectively, and$\varepsilon$is the residual.

To avoid expanding the birth cohort and reducing the temporal precision of describing the risk of incidence and mortality, this study used age-specific data from 2005, 2010, and 2015 for the simulation of the age-period-cohort model [29].

### Statistical analysis

Joinpoint regression analysis was performed using the Joinpoint Regression Program 4.9.1.0 software developed by the National Cancer Institute. The age-period-cohort model was conducted using an online web analysis tool developed by Rosenberg and Check et al. [30]. A statistically significant difference was considered at *P* < 0.05.

## Results

### Incidence of leukemia and its trends in China, 2005–2017

From 2005 to 2017, the total number of new cases of leukemia in the Chinese cancer registry were 144,997, including 82,499 cases in males (56.90%) and 62,498 cases in females (43.10%). A total of 83,343 cases (57.48%) were in urban areas and 61,654 cases (42.52%) were in rural areas. The overall incidence rate was 5.92/100,000 (6.65/100,000 males and 5.18/100,000 females; 6.29/100,000 urban and 5.49/100,000 rural) and the ASIR was 4.54/100,000 (5.14/100,000 males and 3.95/100,000 females; 4.75/100,000 urban and 4.18/100,000 rural). The incidence rate and ASIRs are higher in males and urban areas than that in females and rural areas, respectively. (Table 1).

**Table 1:** Incidence and mortality of leukemia in China, 2005–2017 (1/100,000)

Indexes	National				Male				Female				Urban				Rural		
	Cases	Rates	ASIR/ASMR		Cases	Rates	ASIR/ASMR		Cases	Rates	ASIR/ASMR		Cases	Rates	ASIR/ASMR		Cases	Rates	ASIR/ASMR
**Incidence**																			
2005	2636	4.80	3.96		1484	5.33	4.31		1152	4.25	3.63		2058	5.06	4.18		578	4.06	3.52
2006	3143	5.28	4.11		1752	5.84	4.62		1391	4.71	3.61		2563	5.50	4.32		580	4.46	3.61
2007	3005	5.02	3.94		1721	5.69	4.54		1284	4.34	3.34		2350	5.27	4.19		655	4.31	3.50
2008	3812	5.76	4.32		2145	6.43	4.83		1667	5.08	3.82		3201	6.14	4.59		611	4.37	3.45
2009	4853	5.68	4.34		2744	6.35	4.95		2109	4.99	3.73		3663	6.37	4.85		1190	4.25	3.41
2010	7017	5.63	4.75		3984	6.32	5.42		3033	4.93	4.09		4875	6.09	4.99		2142	4.80	4.30
2011	7992	5.48	4.57		4539	6.16	5.19		3453	4.79	3.96		5083	5.81	4.64		2909	5.00	4.42
2012	11,259	5.68	4.72		6308	6.28	5.30		4951	5.07	4.19		6222	6.19	4.94		5037	5.16	4.50
2013	13,372	5.90	4.82		7726	6.73	5.55		5646	5.06	4.10		7037	6.31	4.94		6335	5.51	4.69
2014	17,215	5.97	4.85		9786	6.69	5.48		7429	5.23	4.23		9332	6.48	5.10		7883	5.47	4.60
2015	20,090	6.26	5.00		11,393	7.00	5.64		8697	5.50	4.36		10,155	6.59	5.06		9935	5.96	4.93
2016	23,443	6.14	4.85		13,450	6.95	5.53		9993	5.32	4.18		12,441	6.46	4.91		11,002	5.82	4.79
2017	27,160	6.22	4.83		15,467	6.99	5.51		11,693	5.43	4.16		14,363	6.74	5.05		12,797	5.74	4.62
Total	14,4997	5.92	4.54		82,499	6.65	5.14		62,498	5.18	3.95		83,343	6.29	4.75		61,654	5.49	4.18
**Mortality**																			
2005	2069	3.77	2.71		1200	4.31	3.15		869	3.21	2.29		1562	3.84	2.61		507	3.56	2.95
2006	2438	4.09	2.88		1385	4.61	3.26		1053	3.56	2.49		1920	4.12	2.79		518	3.98	3.17
2007	2416	4.04	2.80		1381	4.57	3.30		1035	3.50	2.29		1845	4.14	2.73		571	3.76	3.00
2008	2642	3.99	2.71		1462	4.39	2.98		1180	3.60	2.44		2158	4.14	2.72		484	3.46	2.68
2009	3661	4.28	2.88		2161	5.00	3.43		1500	3.55	2.34		2619	4.56	2.91		1042	3.72	2.82
2010	4955	3.98	3.12		2865	4.54	3.67		2090	3.39	2.59		3309	4.14	3.08		1646	3.69	3.16
2011	5768	3.96	3.06		3369	4.57	3.62		2399	3.33	2.53		3760	4.30	3.09		2008	3.45	2.96
2012	8027	4.05	3.14		4692	4.67	3.72		3335	3.41	2.59		4435	4.42	3.16		3592	3.68	3.08
2013	9143	4.04	3.10		5263	4.58	3.60		3880	3.48	2.62		4767	4.27	3.06		4376	3.81	3.11
2014	11,114	3.86	2.88		6455	4.42	3.36		4659	3.28	2.41		5891	4.09	2.81		5223	3.62	2.92
2015	12,850	4.00	2.97		7485	4.60	3.48		5365	3.39	2.48		6507	4.22	2.99		6343	3.80	2.96
2016	15,382	4.03	2.95		8952	4.62	3.46		6430	3.42	2.44		8205	4.26	2.94		7177	3.80	2.94
2017	15,989	3.66	2.65		9274	4.19	3.11		6715	3.12	2.20		8166	3.83	2.62		7823	3.51	2.67
Total	96,454	3.94	2.91		55,944	4.51	3.40		40,510	3.35	2.44		55,144	4.09	2.89		41,310	3.68	2.96

Note: ASIR, age-standardized incidence rate; ASMR, age-standardized mortality rate.

**Fig. 1:**
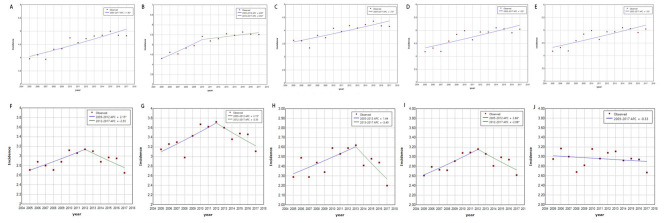
Joinpoint regression in the incidence and mortality of leukemia in China, 2005–2017. Incidence: **(A)** national; **(B)** male; **(C)** female; **(D)** urban; **(E)** rural. Mortality: **(F)** national; **(G)** male; **(H)** female; **(I)** urban; **(J)** rural

The ASIR of leukemia increased from 3.96/100,000 in 2005 to 4.83/100,000 in 2017, AAPC was 1.9% (95% CI: 1.3%, 2.5%). For males, the ASIR of leukemia increased from 4.31/100,000 in 2005 to 5.51/100,000 in 2017, AAPC = 2.2% (95% CI: 1.5%, 3.0%), where the APC for 2005 to 2010 was 4.1% (95% CI: 2.4%, 5.8%), and for 2010 to 2017 was 0.9% (95% CI: 0.0%, 1.8%). For females, the ASIR of leukemia increased from 3.63/100,000 in 2005 to 4.16/100,000 in 2017, AAPC = 1.7% (95% CI: 1.0%, 2.4%). By areas, the ASIR of leukemia in urban increased from 4.18/100,000 in 2005 to 5.05/100,000 in 2017, AAPC = 1.5% (95% CI: 0.9%, 2.2%), that in rural areas increased from 3.52/100,000 in 2005 to 4.62/100,000 in 2017, AAPC = 3.3% (95% CI: 2.1%, 4.4%). The increase rate of leukemia ASIR was higher in rural areas than that in urban areas (Tables 1 and 2; Fig. 1A-E).

**Table 2:** Trend in incidence and mortality of leukemia in China, 2005 − 2017 (%)

Indexes		National		Male		Female		Urban		Rural
**Incidence**										
Periods		2005–2017		2005–2010		2005–2017		2005–2017		2005–2015
APC (95%CI)		1.9* (1.3, 2.5)		4.1* (2.4, 5.8)		1.7* (1.0, 2.4)		1.5* (0.9, 2.2)		3.3* (2.1, 4.4)
t		7.1		5.7		5.4		5.2		6.4
*P*		< 0.001		< 0.001		< 0.001		< 0.001		< 0.001
Periods				2010–2017						
APC (95%CI)				0.9* (0.0, 1.8)						
t				2.3						
*P*				0.049						
AAPC (95%CI)		1.9* (1.3, 2.5)		2.2* (1.5, 3.0)		1.7* (1.0, 2.4)		1.5* (0.9, 2.2)		3.3* (2.1, 4.4)
t		7.1		5.9		5.4		5.2		6.4
*P*		< 0.001		< 0.001		< 0.001		< 0.001		< 0.001
**Mortality**										
Periods		2005–2012		2005–2012		2005–2013		2005–2012		2005–2015
APC (95%CI)		2.1* (0.4, 3.9)		2.6* (0.4, 4.8)		1.4 (0.0, 2.9)		2.7* (0.9, 4.4)		-0.3 (-1.2, 0.6)
t		2.9		2.7		2.3		3.6		-0.8
*P*		0.020		0.028		0.052		0.007		0.434
Periods		2012–2017		2012–2017		2013–2017		2012–2017		
APC (95%CI)		-2.5 (-5.3, 0.3)		-2.7 (-6.2, 0.9)		-3.4 (-7.5, 0.9)		-2.9* (-5.6, -0.1)		
t		-2.1		-1.7		-1.8		-2.4		
*P*		0.071		0.120		0.102		0.044		
AAPC (95%CI)		0.2 (-1.1, 1.5)		0.3 (-1.3, 2.0)		-0.2 (-1.7, 1.3)		0.3 (-1.0, 1.6)		-0.3 (-1.2, 0.6)
t		0.2		0.4		-0.3		0.5		-0.8
*P*		1.000		1.000		1.000		1.000		0.100

Note: AAPC: Average Annual Percentage Change; APC: Annual Percent Change; *Indicates that the APC is significantly different from zero at the alpha = 0.05 level; *Indicates that the AAPC is significantly different from zero at the alpha = 0.05 level.

### Mortality of leukemia and its trends in China, 2005–2017

From 2005 to 2017, the total number of leukemia deaths in Chinese Cancer Registry were 96,454 patients, of which 55,944 (58.00%) were males and 40,510 (42.00%) were females. The total number of deaths in urban areas were 55,144 (57.17%) and in rural areas were 41,310 (42.83%). The overall mortality rate was 3.94/100,000 (4.51/100,000 males and 3.35/100,000 females; 4.09/100,000 urban and 3.68/100,000 rural) and the ASMR was 2.91/100,000 (3.40/100,000 males and 2.44/100,000 females; 2.89/100,000 urban and 2.96/100,000 rural). Both the mortality and ASMRs were higher for males than that for females. The mortality rate in urban areas is higher than that in rural areas, while the ASMR rate is lower than that in rural areas (Table 1).

The ASMR for leukemia decreased from 2.71/100,000 in 2005 to 2.65/100,000 in 2017, AAPC = 0.2% (95% CI: -1.1%, 1.5%), with 2012 as the turn-around point for an increase followed by a decrease with an APC of 2.1% (95% CI: 0.4%, 3.9%) and − 2.5% (95% CI: -5.3%, 0.3%). For males, the ASMR for leukemia decreased from 3.15/100,000 in 2005 to 3.11/100,000 in 2017, AAPC = 0.3% (95% CI: -1.3%, 2.0%), with an increasing trend from 2005 to 2012 with APC of 2.6% (95% CI: 0.4%, 4.8%) and a decreasing trend from 2012 to 2017 with APC of -2.7% (95% CI: -6.2%, 0.9%). For females, the ASMR for leukemia decreased from 2.29/100,000 in 2005 to 2.20/100,000 in 2017, AAPC=-0.2% (95% CI: -1.7%, 1.3%), with 2013 as the turn-around point for an increase followed by a decrease with an APC of 1.4% (95% CI: 0.0%, 2.9%) and 3.4% (95% CI: -7.5%, 0.9%). Urban areas showed an increased from 2.61/100,000 in 2005 to 2.62/100,000 in 2017, with AAPC of 0.3% (95% CI: -1.0%, 1.6%). Rural areas showed a decreasing trend from 2.95/100,000 in 2005 to 2.67/100,000 in 2017, with AAPC of -0.3% (95% CI: -1.2%, 0.6%) (Tables 1 and 2; Fig. 1F-J).

### Age-period-cohort model

**Fig. 2:**
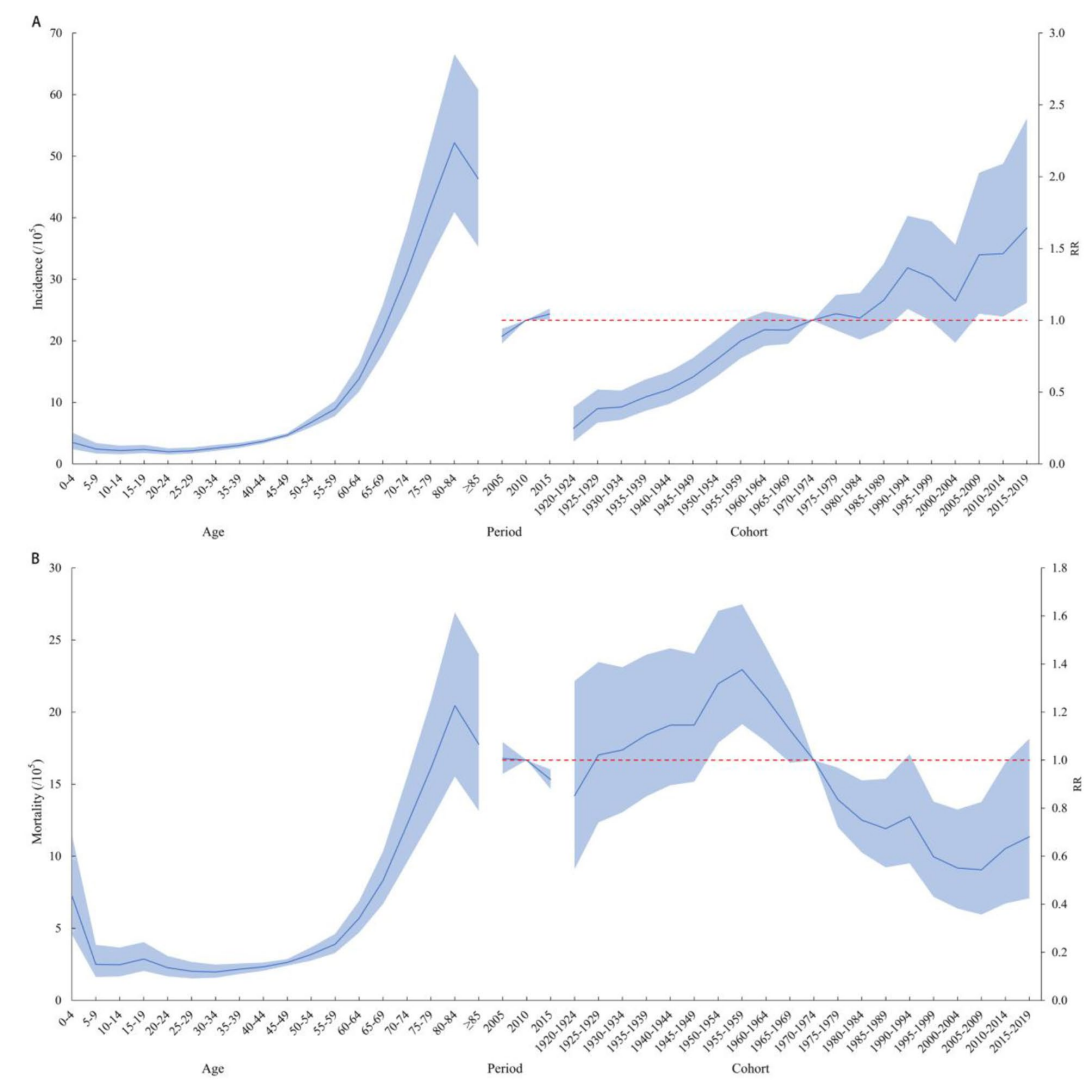
Age-period-cohort model of leukemia incidence and mortality in China, 2005–2017. **(A)** incidence; **(B)** mortality

**Fig. 3:**
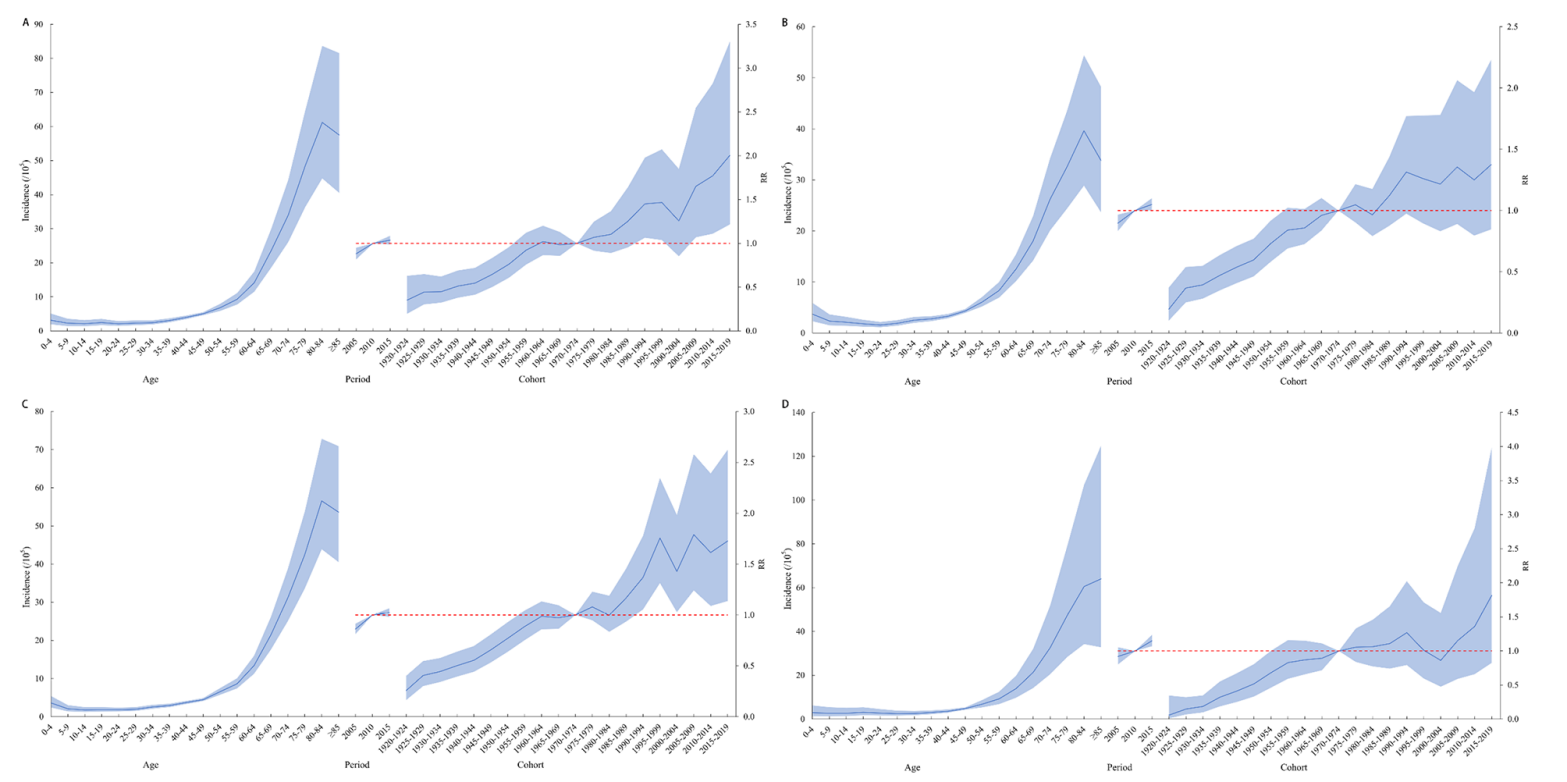
Age-period-cohort model of leukemia incidence in China, 2005–2017. **(A)** Male; **(B)** Female; **(C)** Urban; **(D)** Rural

The trends in age effects on incidence rate were generally consistent across the national, males, females and urban areas. Among those aged 0–49 years, the risk of developing leukemia increased slowly with age. In aged 50–79 years, the risk of developing leukemia all increased rapidly with age, peaking in the 75–79 years. Among those under 18 years, the risk of developing the disease was higher in children aged 0–4 years. In the rural population aged 0–79 years, the age effects all remain consistent with the nation, but the risk of leukemia still tended to increase after the age of 80 years. The trend showed that the contribution of age to the risk of developing leukemia increased gradually with age. In the period effect, the national risk of leukemia incidence gradually increased through time. The cohort effect for the risk of leukemia peaked in 1990–1994 with a relative risk (RR) of 1.37 (95% CI: 0.99, 1.69), then declined slightly in 1995–1999 with an RR of 1.30 (95% CI: 1.08, 1.73), and gradually increased thereafter (Figs. 2A and 3).

**Fig. 4:**
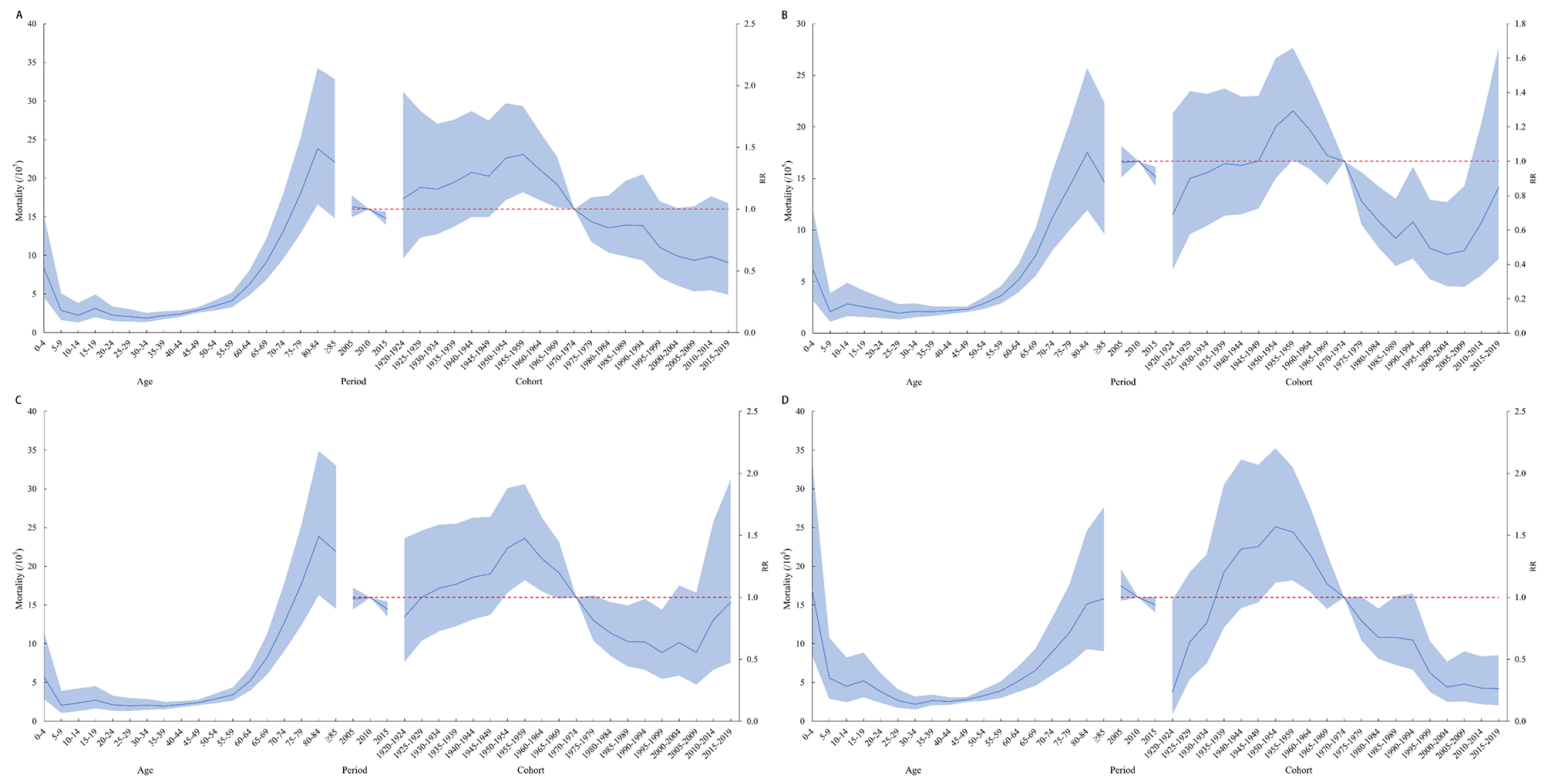
Age-period-cohort model of leukemia mortality in China, 2005–2017. **(A)** Male; **(B)** Female; **(C)** Urban; **(D)** Rural

The trend in the age effects on mortality rate were basically the same for the national, males, females and urban and rural. In the 0–49 age group, the risk of mortality from leukemia is the highest in children aged 0–4 years. Among those aged 50–79 years, the risk of leukemia mortality tended to increase with age. Among the period effects, there was a consistent trend of change for the national, male, female and urban, with a slow decreased trend of leukemia mortality from 2005 to 2017. The risk of rural leukemia mortality with a trend of rapid decline followed by a slow decline, with 2010 as the turning point. The cohort effect for leukemia mortality peaked in 1955–1959 (RR = 1.38, 95%CI: 1.15, 1.65), then decreased and reached its lower point in 2005–2009 (RR = 0.55, 95% CI: 0.36, 0.83) (Figs. 2B and 4).

## Discussion

This study aimed to analyze the epidemiological characteristics of leukemia in China from 2005 to 2017 and estimate their age-period-cohort effects. The results displayed that the ASIR of leukemia from 2005 to 2017 showed an overall increasing trend, and the ASMR showed an increasing trend followed by a decreasing trend with 2012 as the dividing line. The ASIRs and ASMRs of leukemia in males were not only higher than those in females, but also increased faster than in females. The incidence of leukemia was lower in the rural than in urban areas, but the AAPC was 2.2 times higher than urban areas. The risk of leukemia incidence and mortality was higher in children aged 0–4 years. The cohort effect results showed that those born in 2015–2019 had the highest risk of developing leukemia.

The leukemia incidence in China was generally at a moderate level compared to other countries and regions worldwide [31]. The ASIR in China in 2017 was 4.83/100,000, which is lower than the estimated global leukemia incidence rate of 8.32/100,000, 7.10/100,000 in Asia, 8.62/100,000 in the United States based on the latest GLOBOCAN 2020 data, and higher than the 3.98/100,000 in Africa and 3.12/100,000 in India. The ASMR in China was 2.65/100,000 in 2017, lower than the global rate of 4.32/100,000, 3.68/100,000 in Asia, 5.22/100,000 in the United States, and higher than 2.44/100,000 in Africa and 2.43/100,000 in India [32]. This may be related to living behavior in different countries/regions, different ethnicity, geographical location, and the input of medical resources [33] For example, leukemia incidence and mortality rates are relatively higher in the United States compared to China. In terms of dietary structure, the United States is typically known for its high consumption of processed foods and high-calorie meals, including foods high in fat, sugar, and salt [34]. The popularity of fast-food restaurants and chain restaurants in the United States has also made it easier for people to access these high-calorie foods [35, 36]. This dietary pattern is significantly related to the occurrence of obesity [37]. According to the latest data from the United States Centers for Disease Control and Prevention, the adult obesity rate in the United States was about 42.4% [38], significantly higher than in other countries or regions. Previous study showed that a high body mass index (BMI) increased the risk of leukemia, especially chronic lymphocytic leukemia and myeloid leukemia [39]. High BMI significantly increases the prevalence of leukemia, with each 5 kg/m^2^ increase in BMI associated with a 13% increased risk of leukemia [40]. High BMI may affect the occurrence and prognosis of leukemia through a variety of pathways, such as causing a chronic low inflammatory state which can lead to impaired immune system function, thereby increasing the incidence and mortality of leukemia. In addition, the high incidence of childhood leukemia in China may also be related to obesity. A study in the Lancet pointed out that China is the fastest growing country in the world in terms of fast food consumption, and its total consumption is catching up with Western countries [41]. However, the entry and continuous popularity of Western fast food into China has led to a significant increase in children’s weight, because these fast foods with high sugar and fat are more popular among children [42]. This is consistent with another cross-sectional study conducted in China, it found that children with higher BMI consumed fast food more frequently [43]. The total ASIR of leukemia in China showed an increasing trend from 2005 to 2017. Improvements in medical testing and diagnostic techniques, such as the use of genetic testing and deep sequencing, and the standardization of diagnostic criteria, have allowed for a more correct diagnosis of leukemia. Moreover, the change in lifestyle, diet structure, coupled with the progress of urbanization and industrialization, increased exposure to environmental pollution and other risk factors have led to an increase in the incidence of leukemia [5, 44]. The high production and wide range of uses of benzene in China, the world’s largest consumer of pure benzene [45], render it more exposed to the population. Numerous studies have shown that benzene exposure increases the risk of leukemia [46, 47]. Biologically, this relationship also held up, with animals and in vitro experiments found that the target organ of benzene was the bone marrow and that its toxic metabolites can attack hematopoietic stem cells in a variety of ways, causing hematologic toxicity [48]. Benzene can also react with DNA, causing DNA damage and chromosomal abnormalities, including genetic mutations and chromosomal aberrations, which can lead to leukemia [49].

The results of this study showed that leukemia incidence and mortality rates were higher in males than in females. Gender differences in leukemia disease burden can be observed, which was consistent with the findings of previous studies [50, 51]. This may be related to greater exposure of males to risk factors such as environmental pollution, smoking, and unhealthy lifestyles. Studies in countries such as the United States and Canada have shown that residents living in industrial areas have a higher risk of developing leukemia than those living away from industrial areas [52, 53]. Some studies showed that chemicals in cigarettes may cause damage to stem cells and blood-forming cells in the bone marrow, thus increasing the risk of leukemia. In addition, smoking may also increase the risk of developing leukemia by interfering with the normal function of the immune system [54, 55]. The disease burden of leukemia caused by smoking factors in China is the most serious among the four risk factors for leukemia collected by Global Burden of Disease 2019 [5]. And according to the Chinese Center for Disease Control and Prevention Adult Tobacco Survey report, the current smoking rate in China is 52.1% in males and 2.7% in females [56], and the much higher smoking rate in males than in females is also an important factor [57]. This suggests that males are one of the key populations for leukemia prevention and control. The government should regularly conduct health education for males, strengthen tobacco control, and improve occupational work environments. For example, there is still a discrepancy between the current status of implementation of tobacco control measures in China and the requirements of the WHO’s MPOWER measures [58]. Therefore, policies such as national legislation banning smoking in public places, inclusion of smoking cessation medications in medical insurance, a total ban on tobacco advertising, promotion and sponsorship, and an increase in the tax rate and price of tobacco products (with a view to achieving a cigarette retail tax rate greater than or equal to 75% as recommended by WHO) should be introduced and improved as soon as possible [59].

The incidence of leukemia in rural areas was lower than that in urban areas, but the AAPC was 2.2 times higher than urban areas. The accelerated urbanization and dramatic increase in population have increased urban environmental pollution (e.g., air pollution), which may explain the higher incidence in urban areas. This is because air pollution is a risk factor for leukemia development [60]. As shown by the national ecological quality profile in 2020, the substandard rate of urban air pollution in China was as high as 40.1% [61]. However, in recent years, large-scale infrastructure construction and gradual implementation of industrialization policies in rural areas have led to accelerated industrialization and a gradual increase in air pollution. According to China’s National Development and Reform Commission, development zones established on the basis of administrative units at the county level and below has become a new element of rural industrial development. By the end of 2002, there were 6,866 such development zones in China, with a planned area of 38,600 square kilometers, while the area of 75 urban built-up areas by the end of 2020 was only 30,500 square kilometers [62]. China’s rural areas are currently facing the most representative air pollution problems. With industrialization, the rural economy continues to develop well, and different forms of modern equipment are applied to rural production and life, leading to an increasing number of automobile exhaust and production waste gas, which accelerates the decline of air quality [63]. These may be the reasons for the rapid increase in the incidence of leukemia in rural areas.

The risk of leukemia incidence and mortality was highest in children aged 0–4 years among those under 18 years. The cohort effect results also showed that those born in 2015–2019 had the highest risk of developing leukemia. Childhood leukemia is the most common malignancy in childhood and should be of concern to the public, with emphasis on prevention. Prevention based on environmental factors is a very important part of the prevention and treatment of childhood leukemia. A number of studies have confirmed that the yearly increase in the incidence of childhood leukemia is associated with environmental exposures, including radiation, air pollution, chemical exposure, traffic fumes, tobacco, etc. [64]. More than 90% of the world’s population breathes dangerously high levels of air pollutants. A meta-review of the relationship between leukemia and air pollution found that traffic-related air pollution was associated with excess risk of childhood leukemia. The dose-response analysis indicated that in the highest levels, traffic indicators near the child’s residence, traffic density and NO_2_, there may be associated with excess risk of childhood leukemia [65]. Another meta review of maternal exposure to air pollution and risk of leukemia at different times showed that exposure to benzene in the third trimester, as well as exposure to NO_2_ in the second trimester and entire pregnancy, could also increase the risk of leukemia [48]. This prompts the necessity of developing policies aimed at reducing air pollution exposure and protecting special populations to further reduce risks due to air pollutants. For example, when planning homes, schools or other facilities for children, policy makers should consider their distance from main roads, choose green building materials and furniture, such as solid wood flooring and formaldehyde-free panels, and use low volatile and environmentally friendly paints to reduce the volatile harmful substances. Moreover, not only children, but also pregnant women should strengthen their pregnancy health care and raise awareness of maternal health protection to reduce exposure to relevant risk factors. In addition to environmental pollution and lifestyle influencing factors, study showed a correlation between leukemia and genetic factors [66]. A study from Switzerland also found that a family history of oncology was a risk factor for childhood leukemia. When an adult family member is diagnosed with chronic lymphocytic leukemia, the risk of acute lymphoblastic leukemia in children is increased 1.40 times [67]. This suggests that people with a family history of related cancers should undergo more frequent screening, monitoring and diagnostic workups under the guidance of a physician.

The results of the period effect for leukemia showed a trend of increased incidence and decreased mortality. And cohort effect showed a high risk of incidence but a relatively low risk of mortality in the late-birth cohort. These changes may be due to the advances in medical technology and improved medical treatments for leukemia in China, which have made the treatments of leukemia more effective. At present, chemotherapy, hematopoietic stem cell transplantation and targeted therapy are effective treatments for leukemia in China. Among them, with the research of biological targets for leukemia, the “Shanghai program”, “Beijing program” and chimeric antigen receptor T cell therapy innovation have been widely used, and Chinese leukemia has entered the era of precision targeted therapy. Leukemia patients are expected to have a 5-year survival rate of 60-90% in China [68]. It is evident that advances in leukemia treatment have brought it into the era of slow disease management, and lethality has abated. However, the incidence is still rising due to increasing exposure to leukemia risk factors, consistent with another study [60]. These were consistented with the cohort effect in this study, where those in the late birth cohort (birth period was 2005–2017) were synchronized with these changes described above.

Limitations exist in this study. The leukemia data were obtained from the Chinese Cancer Registry Annual Report, 2008–2020. The original data were obtained from the national cancer registry rather than random sampling, so the representation and extrapolation results for the whole population are inadequate. This study also has limitations in terms of timeliness, as there is generally a 3-year time delay in the latest cancer registry data. There are certain limitations to the representativeness and comparability of the data. The subtypes of leukemia were not studied in this study due to insufficient available data.

## Conclusion

The ASIR of leukemia in China showed an overall increasing trend from 2005 to 2017, while the ASMR showed an increasing trend followed by a decreasing trend, with significant gender and regional differences. Among those under 18 years of age, children aged 0–4 years are more likely to develop leukemia and face a higher risk of mortality. This suggests that people should remain to attach importance to the prevention and treatment of leukemia, to strengthen education. It also strengthens the identification and screening of high-risk groups according to gender and age distribution differences, reduces the burden of leukemia disease in the population, improves the healthy living standard of the population, and provides basic data and scientific support for the prevention and treatment of leukemia in China.

## Data Availability

All data relevant to the study are included in the article. It can also available from the China Cancer Registry Annual Report, 2008–2020 published by the National Central Cancer Registry of China.
